# A Framework for the Use and Likelihood of Regulatory Acceptance of Single-Arm Trials

**DOI:** 10.1007/s43441-024-00693-8

**Published:** 2024-09-16

**Authors:** Disha Subramaniam, Colin Anderson-Smits, Rebecca Rubinstein, Sydney T. Thai, Rose Purcell, Cynthia Girman

**Affiliations:** 1 Real World Evidence and Patient Outcomes, CERobs Consulting, Wrightsville Beach, NC USA; 2 Department of Epidemiology, University of North Carolina at Chapel Hill, Chapel Hill, NC USA; 3 Takeda Pharmaceuticals, Cambridge, MA USA

**Keywords:** Single-arm-trials, Uncontrolled-trials, Open-label-studies, External-control-arm

## Abstract

**Background:**

Single-arm clinical trials (SAT) are common in drug and biologic submissions for rare or life-threatening conditions, especially when no therapeutic options exist. External control arms (ECAs) improve interpretation of SATs but pose methodological and regulatory challenges.

**Objective:**

Through narrative reviews and expert input, we developed a framework for considerations that might influence regulatory use and likelihood of regulatory acceptance of an SAT, identifying non-oncology first indication approvals as an area of interest. We systematically analyzed FDA and EMA approvals using SATs as pivotal evidence. The framework guided outcome abstraction on regulatory responses.

**Methods:**

We examined all non-oncology FDA and EMA drug and biologic approvals for first indications from 2019 to 2022 to identify those with SAT as pivotal safety or efficacy evidence. We abstracted outcomes, key study design features, regulator responses to SAT and (where applicable) ECA design, and product label content.

**Results:**

Among 20 SAT-based FDA approvals and 17 SAT-based EMA approvals, most common indications were progressive rare diseases with high unmet need/limited therapeutic options and a natural history without spontaneous improvement. Of the types of comparators, most were natural history cohorts (45% FDA; 47% EMA) and baseline controls (40% FDA; 47% EMA). Common critiques were of non-contemporaneous ECAs, subjective endpoints, and baseline covariate imbalance between arms.

**Conclusion:**

Based on recent FDA and EMA approvals, the likelihood of regulatory success for SATs with ECAs depends on many design, analytic, and data quality considerations. Our framework is useful in early drug development when considering SAT strategies for evidence generation.

**Supplementary Information:**

The online version contains supplementary material available at 10.1007/s43441-024-00693-8.

## Background

Randomized controlled clinical trials (RCTs) are typically the evidentiary basis for the approval by regulatory agencies of new pharmaceutical and device products for use in treating appropriate patients. However, in certain situations, RCTs may not be a feasible study design.

For example, RCTs may not be an option for rare diseases or other conditions where an adequate sample size is hard to obtain globally, let alone regionally [1–3]. RCTs may also not be a feasible study design for debilitating or life-threatening diseases with limited alternative treatment options, as it may be unethical to include a placebo or a significantly less effective comparator. Additionally, RCTs may be impractical in other disease areas with limited alternative treatment options or when early-phase clinical trials for an investigational drug have shown promise, since recruiting and retaining patients for the placebo arm could be challenging. [4]

In such cases, single-arm trials (SATs) are often used to support regulatory submissions for approval of new indications for drugs and biologics [5, 6]. In SATs, a group of individuals with the condition of interest receiving the investigational new drug or biological are followed over time to observe their response to treatment. [7]

There is established precedence for use of SATs in regulatory submissions in the United States and European Union. These include preliminary and early phase studies of product safety, and open-label extensions of randomized Phase 2 and 3 studies [8, 9]. These studies may be submitted as supportive evidence alongside traditionally well-controlled trials such as RCTs.

Under certain limited circumstances, SATs may be submitted as pivotal evidence for determination of efficacy and safety for approval. When serving as the basis for approval, SATs may use an external control arm (ECA) to mitigate methodologic and statistical concerns arising from the lack or inadequacy of an enrolled comparator group [10]. An SAT with an ECA has a “control group that consists of patients who are not enrolled as part of the single-arm trial, i.e., there is no concurrently randomized control group” [11]. External control arm data may come from numerous sources, including past clinical trial data or real-world data (RWD) sources such as registries or natural history studies, electronic health records (EHRs) or administrative claims. [12, 13]

Recent studies have reported widespread use of Real World Evidence (RWE) in FDA submissions and EMA applications for marketing authorization [14, 15]. Regulatory acceptance of submissions using single-arm designs and external control arms has increased, concordant with an more submissions for rare disease and gene therapy products [14, 16]. Applications along with Health Technology Assessments (HTAs) and regulatory agency assessments of SATs have been examined in oncology [17, 18]. However, there are limited resources to guide the design and analysis of non-cancer programs to improve the likelihood of regulatory acceptance. Applications of SATs in non-oncology contexts may have a different regulatory likelihood of acceptance. Yet, studies that examine submissions in rare diseases and other non-oncology indications fail to identify specific methodologic and other features of the intervention and study design that led to regulatory success. [11]

As such, we developed a framework for considerations in SAT strategies and ECAs that may affect likelihood of regulatory success. Our framework helped identify key types of submissions that may face greater regulatory challenges: novel approvals fo first indications. We then reviewed all FDA and EMA approvals from 2019 to 2022 that used SATs as pivotal evidence for first indications of new molecules and biologicals to identify and understand the common factors associated with regulatory acceptance. Since SAT and ECA approaches are documented within oncology our review focused on non-oncology approvals for first indications. [18, 19]

## Methods

### Development of Framework

We developed a framework to understand the regulatory acceptability of an SAT strategy in multiple phases, drawing from a narrative literature review, interviews across disciplines of drug development, and the extensive experience of our core team, who have over 40 years of experience in drug development and regulation. Systematic phased focus groups were conducted in in a large pharmaceutical company with senior leaders in epidemiologiy, statistics, regulatory affairs, clinical science and clin pharmacology. The first focus groups probed as to possibilities of where single arm studies could be used, supplemented with a narrative review of the literature and regulatory guidance documents. This was followed by focus groups targeting different medical and regulatory considerations that could impact potential acceptance of SAT.

**Figure 1:**
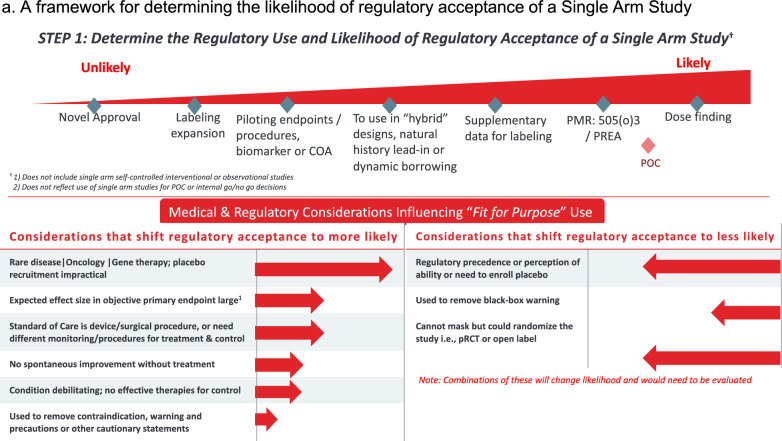
A framework for determining the likelihood of regulatory acceptance of a Single Arm Study. Footnote: Top of figure shows factors regulatory decisions believed to be less likely (on left) for regulatory acceptance of a single arm trial (SATs), with increasing likelihood for decisions moving to the right of graph. Bottom left part of figure reflects considerations that may increase the likelihood of regulatory acceptance of SATs, while the right side shows those that may decrease likelihood of SAT acceptance, depending on regulatory decision. The scope of this study is novel approvals, indicated on the far left end of the spectrum.

**Figure 2:**
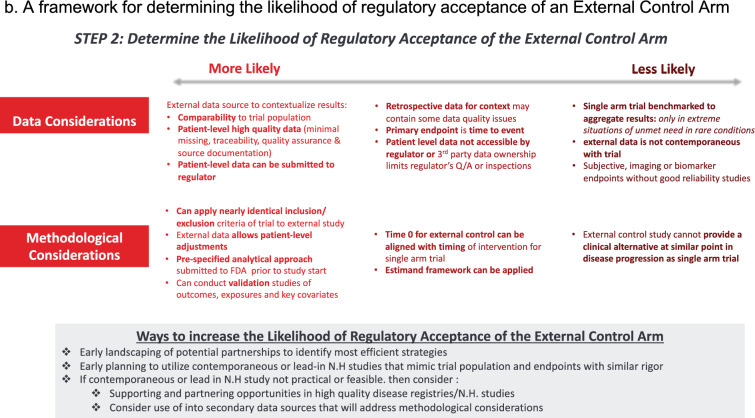
A framework for determining the likelihood of regulatory acceptance of an External Control Arm. Footnote: Data considerations and methodological considerations that are less likely (left of graph) or more likely (right of graph) to lead to regulatory acceptance of an external control arm.

Our framework differentiates between the diverse types of SATs, including supportive SATs such as pediatric extrapolations and SATs submitted as pivotal evidence. The framework used to identify the types of submissions that we expected to face the most regulatory challenge, which informed the scope of our study. As a first test of the framework, we applied it to a subset of approvals from 2019 to 2022 to understand regulatory responses to *novel submissions outside of oncology*. Reviewing responses to other aspects of the framework were not in scope for this study. We then used the regulatory and medical considerations listed in Fig. 1 of the framework and the data and methodological considerations listed in Fig. 2 of the framework to guide the key outcomes for abstraction.

### Selection of FDA Approvals and EMA Authorisations

We identified all FDA approvals and EMA authorisations from 2019 to 2022 for which at least one Phase 2 or 3 SAT was submitted as pivotal evidence.. FDA CDER approvals were identified from the Compilation of CDER NME and New Biologic Approvals 1985–2022 from Drugs@FDA website and CBER approvals were identified from the Biological Approvals by Year page on the CDER Website. EMA authorisations were identified from the table of European public assessment reports (EPARs) for all human and veterinary medicine, automatically excluding all veterinary products and products for which authorization status was not “Authorised”.

Using the information on indication available in the EMA and FDA databases, products were screened to exclude any oncologic and similar indications like radiologic, non-malignant tumors, pre-cancer indication. To keep review focus predominantly on therapeutics, we excluded blood products like blood typing reagents, molecules designed for imaging, and diagnostic assays. Finally, we excluded vaccines as a special case.

Four products for which reviews described a single-arm trial as pivotal (Oxlumo, Xenpozyme, Oxbryta, and Voxzogo) were excluded, because the purpose of these SATs was to extrapolate efficacy and safety data to pediatric populations at the same time as the original submission for approval. Although pediatric extrapolation studies are included in the framework, they did not meet the criteria for this review, which examined only the original approval for a first indication.

### Identification of Submissions with Single-Arm Trials

Individual review documents for each product with a relevant indication were examined to determine whether at least one phase 2/3 or phase 3 SAT was included in the submission. For FDA approvals, clinical and statistical review documentation were reviewed where available. Otherwise, integrated or multi-discipline review documentation were used. For EMA authorisations, the “Clinical aspects”, “Clinical efficacy”, and “Clinical safety” sections of the product’s EPAR were reviewed. In documents for both agencies, where available, any comprehensive table of clinical studies submitted was examined to determine if the body of evidence submitted for review included any Phase 2 or 3 SAT. In cases of ambiguous study design or phase, any study cited in the approval documentation was cross-referenced with results from a search of Clinicaltrials.gov, confirming that a study was a SAT if the listed intervention model was “Single-group assignment”.

### Exclusion of Submissions with Solely Supportive Single-Arm Trials

Approvals identified to have included SATs were then assessed to determine whether the SAT was used as pivotal or supportive evidence. In documents for both agencies, any comprehensive table of clinical studies submitted was examined. While all FDA and EMA approvals presented table(s) of clinical studies used for the approval, they differed in how the review presented pivotal vs. supportive evidence. In some cases, review documents included tables that specified “pivotal” or “primary” evidence over supportive evidence. In these cases, any study not listed as supportive was considered pivotal. In other cases, the review documents described in words in the review strategy which trials were purely supportive evidence versus pivotal. If there was only one Phase 2 or 3 trial listed at all for efficacy, it was considered pivotal. Where possible, explicit text was used from approval documentation that described each SAT as either pivotal or supportive. Otherwise, if the study was described in the approval documentation or Clinicaltrials.gov as an open-label extension (OLE) or long-term extension (LTE) of a controlled trial, it was classified as supportive or non-pivotal evidence. If the study was ongoing at the time of submission, it was classified as non-pivotal evidence unless the review text explicitly stated that an ongoing study with a pre-specified data cutoff point was used as pivotal evidence. Finally, submissions for which a pediatric extrapolation study was submitted at the same time as the submission for the first indication in an adult population were excluded.

A primary reviewer identified SATs in FDA and EMA submissions. An additional reviewer cross-checked between the FDA and EMA approvals of the same products to assess any discrepancies.

### Document Search and Data Abstraction

Regulatory documents were evaluated using a pre-specified template developed from our framework for data abstraction. Data from the same regulatory documents were used to identify single arm trials (clinical and statistical review documents for FDA approvals and EPAR documentation for EMA authorisations). Again, for FDA approvals, if clinical and statistical review documents were not available for data abstraction, clinical sections of integrated or multi-discipline reviews were used. Key information abstracted included:

1. General submission and approval information, including details on product and indication, agency and center (if applicable), date of approval or authorization, and any orphan and/or priority designation
2. Information on totality of pivotal evidence submitted (i.e. whether relevant SAT/SATs were sole pivotal evidence or submitted alongside other traditionally well-controlled studies)
3. Agency reviewer responses (critiques and positive assessments) to submission of pivotal SAT(s), including methodological or statistical issues and any information on how therapeutic context influenced acceptability of study design; corresponding to Step 1 of the framework (Fig. 1)
4. Information on external control arms, including data source for control arm and details on RWD used, if applicable
5. Agency reviewer responses (critiques and positive assessments) to submission of external control arm, if applicable, including methodological or statistical issues and any information on how therapeutic context influenced acceptability of study design; corresponding to Step 1 of the framework (Fig. 2)
6. Labeling information, including whether SAT and/or external control arm (where applicable) was mentioned in product labeling (FDA) or package leaflet (EMA)

A full list of abstracted fields and some variable definitions are included in Supplementary Appendix Table A2.

## Results

**Figure 3:**
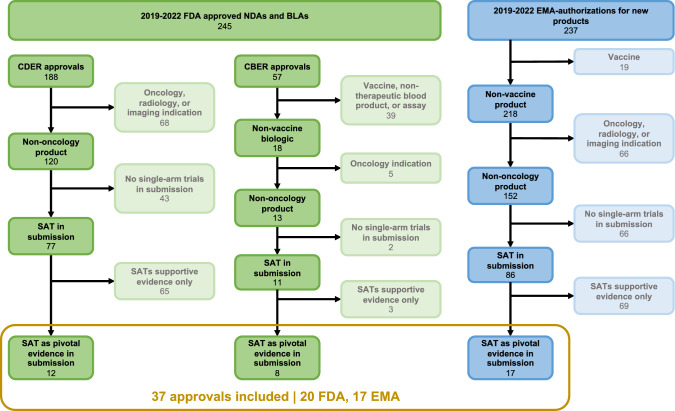
Inclusion and exclusion criteria flowchart for selection of 37 FDA and EMA approvals into final analysis.

Of 482 FDA and EMA product approvals from 2019 to 2022, 37 approvals—20 FDA and 17 EMA—were identified as non-oncology approvals that included a pivotal SAT in the submission. (Table 1; Fig. 3). For these 37 approvals, we abstracted data for 101 fields (Supplementry Appendix Table A2). In both FDA and EMA approvals, the majority of applicants utilized SATs as the sole pivotal efficacy evidence (Table 2). Characteristics of the SATs were largely similar between FDA and EMA approvals, except for the inclusion of SATs in patient-facing product labeling. In FDA approvals, 18/20 (80%) of FDA-approved applications mentioned the use or findings from the pivotal SATs in product labels for clinicians and patients. These approvals were for the drugs Amvuttra, Pyrukynd, Enjaymo, Nextstellis, Nulibry, Imcivree, Zokinvy, Pretomanid, Vyondys 53, Egaten, Skysona, Zynteglo, Rethymic, Ryplazim, Xembify, Zolgensma, Asceniv, and Esperoct. Meanwhile, only 1 of the 19 EMA-approved applications (5%), Esperoct, mentioned a SAT as evidence in the package leaflet.

**Table 1:** Summary of Included Products and Key Characteristics

Product Name (Brand)	INN	Drug or Biologic	Indication	Rare Disease	Gene Therapy	FDA			EMA		
						Date of Approval (Gray if Not FDA Approved in Study Period)	Orphan Designation	Priority Review	Date of Authorization (Gray if Not EMA Approved in Study Period)	Orphan Designation	Accelerated Assessment
Camzyos [20]	mavacamten	Drug	Adults with (NYHA) class II-III obstructive hypertrophic cardiomyopathy (HCM)	Yes	No	4/28/2022	Yes	Yes			
Amvuttra [21, 22]	vutisiran	Drug	Polyneuropathy of hereditary transthyretin-mediated amyloidosis in adults	Yes	No	6/13/2022	Yes	No	9/15/2022	Yes	No
Pyrukynd [23, 24]	mitapivat	Drug	Hemolytic anemia in adults with pyruvate kinase (PK) deficiency	Yes	No	2/17/2022	Yes	Yes	11/9/2022	Yes	No
Enjaymo [25, 26]	sutimlimab	Drug	Hemolysis in adults with cold agglutinin disease (CAD)	Yes	No	2/4/2022	Yes	Yes	11/15/2022	Yes	no
Nextstellis [27]	etonorgestrel and estetrol	Drug	Females of reproductive potential to prevent pregnancy	No	No	4/15/2021	No	No			
Nulibry [28, 29]	fosdenopterin	Drug	Molybdenum cofactor deficiency (MoCD) Type A	Yes	No	2/26/2021	Yes	Yes	9/15/2022	Yes	No
Imcivree [30, 31]	setmelanotide	Drug	Obesity due to proopiomelanocortin (POMC) or proprotein convertase subtilisin/kexin type 1 (PCSK1) deficiency	Yes	No	11/25/2020	Yes	Yes	7/16/2021	Yes	No
Zokinvy [32, 33]	lonafarnib	Drug	Processing-deficient Progeroid Laminopathies with either heterozygous LMNA mutation with progerin-like protein accumulation or ZMPSTE24 mutations	Yes	No	11/20/2020	Yes	Yes	7/18/2022	Yes	No
Dojolvi [34]	triheptanoin	Drug	Pediatric and adult patients with long-chain fatty acid oxidation disorders (LC-FAOD)	Yes	No	6/30/2020	Yes	No			
Pretomanid/Dovprela [35, 36]	pretomanid	Drug	Pulmonary extensively drug resistant (XDR) or treatment-intolerant or nonresponsive multidrug-resistant (MDR) tuberculosis (TB)	No	No	8/14/2019	Yes	Yes	7/31/2020	Yes	No
Vyondys 53 [37]	golodirsen	Drug	Duchenne muscular dystrophy (DMD) with amenable to exon 53 skipping	Yes	No	12/12/2019	Yes	Yes			
Egaten [38]	triclabendazole	Drug	Fascioliasis in patients 6 years of age and older	No	No	2/13/2019	Yes	Yes			
Skysona [39]	elivaldogene autotemcel	Biologic	Boys 4–17 years of age with early, active cerebral adrenoleukodystrophy (CALD)	Yes	Yes	16-Sep-22	Yes	Yes			
Zynteglo [40]	betibeglogene autotemce	Biologic	ß-thalassemia	Yes	Yes	19-Aug-22	Yes	Yes			
Rethymic [41]	allogeneic processed thymus tissue–agdc	Biologic	Congenital athymia	Yes	No	10/8/2021	Yes	Yes			
Ryplazim [42]	plasminogen, human-tvmh	Biologic	Plasminogen deficiency type 1 (hypoplasminogenemi)	Yes	No	6/4/2021	Yes	Yes			
Xembify [43]	immune globulin subcutaneous human-klhw	Biologic	Primary Humoral Immunodeficiency (PI) in patients 2 years of age and older	Yes	No	7/3/2019	No	No			
Zolgensma [44, 45]	Onasemnogene abeparvovec	Biologic	Spinal muscular atrophy (SMA) with bi-allelic mutations in the survival motor neuron 1 (SMN1) gene	Yes	Yes	5/24/2019	Yes	Yes	5/18/2020	Yes	No
Asceniv [46]	mmune globulin intravenous, human – slra	Biologic	Primary humoral immunodeficiency in adults and adolescents (12 to 17 years of age)	Yes	No	4/1/2019	No	No			
Esperoct [47, 48]	turoctocog alfa pegol	Biologic	Adults and children with hemophilia A	Yes	No	2/19/2019	No	No	6/20/2019	No	No
Mycapssa [49]	betibeglogene autotemce	Drug	Adult patients with acromegaly who have responded to and tolerated treatment with somatostatin analogues	Yes	No				12/2/2022	Yes	No
Roctavian [50]	valoctocogene roxaparvovec	Biologic	Severe haemophilia A (congenital factor VIII deficiency) in adult patients without a history of factor VIII inhibitors and without detectable antibodies to adeno-associated virus serotype 5 (AAV5).,	Yes	Yes				6/23/2022	Yes	No
Upstaza [51]	eladocagene exuparvovec	Biologic	L amino acid decarboxylase (AADC) deficiency with a severe phenotype	Yes	Yes				5/19/2022	Yes	No
Voraxaze [52]	glucarpedase	Biologic	Adults and children (aged 28 days and older) with delayed methotrexate elimination or at risk of methotrexate toxicity	No	No				11/11/2021	Yes	No
Lydisilka [53]	drospirenone and estestrol	Drug	Oral contraception	No	No				3/25/2021	Yes	No
Libmeldy [54]	atidarsagene autotemcel	Biologic	Metachromatic leukodystrophy (MLD) characterized by biallelic mutations in the arysulfatase A (ARSA) gene	Yes	Yes				10/15/2020	Yes	No
Idefirix [55]	imlifidase	Biologic	Highly sensitised adult kidney transplant patients with positive crossmatch against an available deceased donor	Yes	No				6/25/2020	Yes	No
Cufence [56]	trientine dihydrochloride	Drug	Wilson’s disease in patients intolerant to D-Penicillamine therapy	Yes	No				5/29/2019	Yes	No

**Table 2:** Summary of Characteristics of Pivotal Evidence in Included Submissions

Characteristic of Pivotal Evidence	N (%)—FDA (N = 20)	N (%)—EMA (N = 17)
Single-arm trial(s) submitted as pivotal evidence alongside 1 or more traditionally controlled trials (randomized controlled or open-label parallel assignment trials)	8 (40)	7 (41)
Single-arm trial submitted as sole pivotal efficacy evidence in submission	12 (60)	10 (59)
Pivotal single-arm trial was Phase 2*	8 (40)	5 (29)
Pivotal single-arm trial was Phase 3*	15 (70)	13 (77) +
Single-arm trial and/or external control arm was included in product labeling, package insert, or summary of product characteristics	18 (90)	2 (12)

*Three studies were considered phase 2/3

+ One study was considered phase 2/3

Two approvals, Amvuttra (EMA and FDA), and Egaten (FDA), were atypical in their uses of single-arm trial data. Amvuttra was approved by the FDA and EMA in 2022 to treat polyneuropathy of hereditary transthyretin-mediated amyloidosis in adults. While this application included a randomized controlled trial, pivotal efficacy evidence functionally came from a single-arm trial, since only investigational arm of the RCT was compared to a historical control arm from a previous trial for the primary endpoint analysis [21, 22]. Egaten was approved by the FDA in 2019 to treat fascioliasis in patients 6 years of age and older. One randomized controlled trial was submitted for this product; however, a single-arm trial compared to a historical control arm was also used as pivotal evidence. One arm of a study evaluating two randomized arms of different doses of the experimental medication was compared to a an active control arm of a different past trial. Determination of efficacy was made by the totality of pivotal evidence, which included single-arm data with historical control [38]. In each situation, one investigational arm of an RCT was compared to a historical control arm to generate pivotal efficacy evidence, so both Amvuttra and Egaten were included.

Characterization of ECA and comparator arms for each application was conducted. (Table 3). Real-world-data (RWD) ECAs, were utilized by a sizable proportion of both FDA (45%) and EMA-approved (47%) applicants. Strikingly, no applications used exclusively claims data or EHRs as a form of RWD in an ECA. Instead, all of the RWD ECAs were based on registries or natural history (NH) controls. Some of these NH studies could have utilized EHRs to populate case report forms. A similar proportion of FDA and EMA-approved proposals (35%) compared SAT results to a non-patient level aggregate benchmark. The use of baseline-controlled participants, in which participants are compared to their own values prior to intervention, was common in applications submitted to both agencies. The use of a historical control group from a prior controlled trial was much more common in FDA approvals (20%) than EMA approvals (6%).

**Table 3:** Summary of Characteristics of External Controls/Comparators

Type of control or comparator*	N (%)—FDA (N = 20)	N (%)—EMA (N = 17)
Baseline-controlled (participants in single-arm trial compared to their own values prior to intervention in a pre-post design)	8 (40)	8 (47)
RWD-based external control arm	9 (45)	8 (47)
EHR	0 (0)	0 (0)
Natural History Cohort^+^	9 (45)	8 (47)
Claims	0 (0)	0 (0)
Historical control group from prior controlled trial	4 (20)	1 (6)
Benchmark (single-arm data is compared to an aggregate value)	7 (32)	6 (32)

*Some single-arm trials used multiple comparators for context

+ Natural History Cohort sources included prospective cohorts, xretrospective chart reviews, reviews of case reports in the literature, and disease registries

Factors in the aforementioned framework may have contributed to the approvals of applications using SATs as pivotal evidence (Table 4). The most common justifications for approval in this context were medical conditions with an established natural history and no spontaneous improvement (condition progressively deteriorates and does not improve without treatment, seen in over 80% of FDA and EMA approvals), and conditions with either no effective therapies or limited standard or care options (seen in over 80% of FDA and EMA approvals).

**Table 4:** Agency and Regulator Responses to Submission of Pivotal Single-Arm Trial

Feature of reviewer response to submission	N (%)—FDA (N = 20)	N (%)—EMA (N = 17)
Rare disease or gene therapy with inability to recruit placebo	18 (90)	15 (88)
Not rare disease but perceived inability to recruit placebo	2 (10)	1 (5.8)
Objective primary endpoint*	18 (90)	15 (88)
Large expected effect size in primary endpoint	9 (45)	3 (18)
SoC is a device/ surgical procedure/need different procedures for intervention & control	0 (0)	2 (12)
Condition has established natural history without spontaneous improvement	18 (90)	15 (88)
Severe condition with no effective therapies for control/limited SoC options	16 (80)	12 (71)
Intervention could not be masked but could have been randomized e.g., open label parallel or randomized assignment	1 (5)	0 (0)
Intervention requires complex safety assessment or active comparator for safety contextualization	0 (0)	0 (0)
Regulator perception of need to enroll placebo	0 (0)	0 (0)

*Objectivity of endpoint either explicitly noted by regulator or verified by authors as implicit

This phenomenon of approvals in rare conditions with no expected spontaneous improvement and limited standard of care options is exemplified in the EMA approval for the drug Upstaza, indicated for the treatment of patients aged 18 months and older with a clinical, molecular, and genetically confirmed diagnosis of aromatic L amino acid decarboxylase (AADC) deficiency with a severe phenotype (Box 1). [51]

Box 1

“Giv“AADC deficiency is a rare autosomal recessive disorder of dopaminergic and serotonergic pathways... Most patients with AADC deficiency do not develop functional motor movement, fail to achieve motor milestones (e.g., full head control and the ability to sit, stand, and walk), and are at risk of an early death in the first decade of life. Consequently, patients with AADC deficiency require life-long care... No therapies are presently approved for the treatment of AADC deficiency. Existing therapies are primarily intended to treat symptoms and do not treat the underlying cause of the disease... The majority of patients, particularly those with no motor development, do not respond to available treatments because these therapies cannot replace or increase dopamine production in the brain to adequately improve motor function and allow achievement of developmental milestones.”

In nearly 60% of FDA applications, regulatory reviewers noted that it would be challenging to recruit sufficient participants in a placebo group due to the rare nature of the condition. However, this was noted considerably less often in EMA applications (37%). FDA reviewers frequently commented on the inability for investigators to recruit controls, and suggested alternatives such as ECAs. An example was the FDA approval for the drug Amvuttra, indicated for the polyneuropathy of hereditary transthyretin-mediated amyloidosis (hATTR amyloidosis) in adults (Box 2). [21]

Box 2

“Given the life-threatening nature of hATTR amyloidosis and the existence of approved therapies, it would not be ethical to use a concurrent placebo control group in the HELIOS-A study; therefore, it is reasonable to consider the use of external control, if feasible.”

In some cases, regulators noted that an RCT would be difficult to conduct due to a lack of established or effective standard of care (42–45%) in a deteriorating condition. A large effect size in the primary endpoint (45% vs 18%) was noted in more FDA applications compared to EMA, and the objective nature of the primary endpoint was explicitly noted in 36% of FDA vs 21% of EMA applications. For approvals where the regulator did not explicitly make a statement about the objectivity of the primary endpoint (or lack thereof), we directly assessed endpoint objectvity. Our study reviewers determined that in all of these cases endpoints were clearly objective. Example of implicitly objective endpoints included laboratory pharmacokinetic values, weight changes relative to participant baseline, or whether or not a participant received a supportive treatment such as mechanical ventilation during the follow-up period. For these approvals, we determined that regulators primary endpoint objectivity was implied and thus not explicitly stated in the review. We also examined observed effect size in FDA approvals, finding that in a majority (20 of the 22) approvals, a large effect size in the primary endpoint was observed, even if not initially expected. An example of an insufficiently large effect size was seen in the FDA approval for Skysona, a gene therapy indicated to slow the progression of neurologic dysfunction in boys 4–17 years of age with early, active cerebral adrenoleukodystrophy (CALD)) (Box 3). [39]

Box 3

"In summary, although eli-cel was successful on the primary efficacy endpoint, the clinical benchmark value of 50% is not meaningful, in particular because Population #1 had much more severe disease at baseline as compared to the population treated with eli-cel in Study ALD-102, and we do not have an appropriate comparator population to understand what proportion of patients with early, active CALD as defined by the ALD102 eligibility criteria would progress to MFD or death within 24 months of diagnosis in the absence of treatment. Unfortunately, this makes interpretation of the prespecified primary efficacy endpoint difficult, and success on the primary efficacy is not meaningful in the demonstration of eli-cel efficacy as compared to the natural history of disease."

If the reviewers did not explicitly state an expectation or observation of large effect size, we defined a large effect size as an RR > 1.5, in accordance with Temple 2012. [57, 58]

Table 5 describes reviewers’ perceptions of limitations in the use of ECAs in NDAs. Limitations and criticisms of ECAs were considerably more common in FDA applications than EMA applications. A common critique from both agencies (25–29%) was that the ECA based on registry or natural history study could not provide an equivalent clinical endpoint in disease progression as the clinical endpoint in the SAT, making it difficult to estimate the true efficacy of the drug in the single-arm trial. Almost half (45%) of FDA-reviewed applications were criticized for using ECAs that were not contemporaneous to the SAT. Twenty-seven percent of FDA-reviewed applications were flagged for using ECAs with subjective endpoints that lacked reliability and for using ECAs that were not comparable with SATs. Commonly, the investigators failed to achieve covariate balance at baseline between the ECA and SAT participant samples, or they selected ECAs that used different inclusion and exclusion criteria as the SAT, making it hard to determine whether differences in clinical endpoints among the single-arm trial and the ECA arms are due to effects of the drug in the trial, or due to confounding factors. For example, in the aforementioned application for Amvuttra, the FDA reviewers note lack of such comparability (Box 4). [21]

**Table 5:** Agency and Regulator Responses to ECA (Includes Critiques of Any Non-baseline Controlled Pivotal Single-Arm Trial)

Feature of comparator/external control	N (%)—FDA	N (%)—EMA
Benchmark: Single arm trial bench-marked to aggregate (not patient-level) results	5 (25)	1 (5)
Data access: Patient level data not accessible by regulator or 3rd party data, ownership limits regulators QA or inspections;	3 (15)	0 (0)
Endpoint selection: Subjective, imaging, or biomarker endpoints without good reliability studies or time to event endpoint	6 (30)	2 (11)
Timing: ECA not contemporaneous to trial	9 (45)	0 (0)
Setting: ECA not generated among geographically representative populations and/ or similar practice setting as single arm trial	3 (15)	0 (0)
Baseline covariates: ECA and trial arms not balanced on baseline covariates; comparable inclusion/exclusion criteria cannot be applied to both arms	6 (30)	2 (12)
Disease status: cannot provide a clinical alternative at a similar point in the disease progression as the single arm trial	5 (25)	5 (29)
Outcome measurement: risk of outcome ascertainment bias in the comparator	4 (20)	0 (0)
Other Data quality issues	8 (30)	4 (24)

Box 4

“The groups are not very comparable at baseline across studies APOLLO and HELIOS-A on the primary outcome measure, mNIS+7 (among other baseline characteristics as mentioned above). For reference, the standard error of the difference between Vutrisiran-HELIOS-A and Placebo APOLLO is estimated as 5.30 and standard error of the difference of the two Patisiran groups is estimated as 6.98, thus the cross study baseline mean differences are sizeable, even after accounting for their standard error…

The APOLLO placebo and HELIOS-A Vutrisiran arms seem to have several other differences in patient characteristics as well. For example, Race proportions are different (32 vs. 17% Asian, genotype: 52 vs 44% V30M, 75 vs. 65% Male, 74 vs 61 NIS >50, Cardiac subpopulation 47% vs. 29%). Comparison to an external control with such clear differences in patient composition is not likely to be reliable.”

In all scenarios using an external control arm, investigators tried to adjust for differences in covariates by treatment arm, but reviewers often still found them insufficiently balanced (see Box 5 for sample FDA comment for the aforementioned treatment Skysona. [39]

Box 5

“The TPES-103 population had similar comparability issues to the TPES-101 population, namely older age at treatment and higher baseline Loes score compared to the TP-102 population.... The Applicant provided propensity score (PS) adjustments to account for such differences, but we do not believe PS adjustments are sufficient to account for the known and unknown baseline differences between groups. As such, the adjustments were minimal, and therefore results are not shown.”

Many FDA-approved applications also mentioned outcome misclassification and data quality issues. Examples include misclassification of the patient catchment area and changes in study designations from exploratory studies to efficacy-finding studies. In one case, during the review for Esperoct, which is indicated to treat adults and children with hemophilia A, the EMA alerted the FDA of multiple data quality issues, which the FDA later included in their report, regarding clinical inspection findings for sites. EMA stated that they identified significant deficiencies in data quality and integrity, and the rights and safety of patients.

FDA reviewers were explicit in the data quality issues they identified with the application for the aforementioned treatment Skysona (Box 6). [39]

Box 6

“We do not agree that repeat HSCT for failure of initial HSC graft is an outcome equivalent to MFD or death, and therefore do not agree that repeat HSCT should be imputed as failure of MFD-free survival. Taking this and other previously discussed data limitations into account (bias influencing MFD identification, retrospective data collection for part of Study ALD-103, few MFDs and deaths in the overall populations), the KM comparisons between TPES-103 populations and TP-102 as performed by the Applicant are difficult to interpret.”

## Discussion

We reviewed all approvals for first indications for non-oncology applications based on SAT strategies submitted to FDA and EMA to summarize common factors. Briefly, we found that regulatory approvals primarily occurred in contexts involving rare diseases characterized by limited or insufficient standard of care options and a notable unmet medical need in debilitating or life-threatening conditions. When implemented, external control arms (ECAs) most frequently were derived from natural history studies, both retrospective and prospective. Criticisms of ECAs commonly revolved around issues such as an imbalance between the ECA and trial arm, leading to confounding. Additionally, concerns were raised about outcome ascertainment bias resulting from measurement errors or subjective endpoints, along with data quality issues attributed to missing data, potentially introducing selection bias.

Patient-facing labels occasionally referenced single-arm trials, suggesting their relevance in the context of communication to patients. FDA approvals more frequently included information on pivotal single-arm trials and ECAs in product labeling than did EMA approvals. This may be due to differences between the agencies in the provision product information to patients and providers. FDA’s package insert serves as a label for both healthcare professionals and patients, while the EMA provides a separate summary of product characteristics (SmPC) for providers that differs from the patient-facing package leaflet or label [59, 60]. While reviewing EMA SmPC documents was not in scope for this review, previous studies have found these documents to contain detailed information on clinical trials. [61, 62]

External control arms (ECAs), frequently drawn from registries or natural history studies, played a key role providing context to single-arm trials. Notably, our analysis found no ECAs that explicitly described using EHR or claims data. We found that in the absence of these data sources, submissions often relied on natural history studies to provide necessary context and while many NH studies did used patient-level healthcare data through retrospective chart reviews, none mentioned EHR data explicitly. Reviews did not explicitly state which such chart reviews used EHRs or whether EHRs were used to populate case report forms. Verification was not possible due to differing timelines of transition from paper to electronic records across health systems. Nevertheless, our finding is consistent with the documented gap in the availability of research-grade Real World Data (RWD) for the use of external control arms in rare diseases [63]. This scarcity poses a challenge in utilizing external control arms from electronic health records or claims data for non-oncology trials particularly in rare diseases, where they can contribute essential contextual information to the evaluation of single-arm trials. These observations deepen our understanding of the regulatory landscape surrounding single-arm trials and highlight the challenges associated with the choice of external control arms, as well as the reliance on natural history studies for context. As clinical trials increasingly utilize EHR data for various purposes and methodological approaches continue to evolve for implementing EHRs as external control arms, we may see EHRs will see increased use as ECAs in non-oncology indications. [64–66]

The findings of the current study align with several past findings from other reviews of regulatory submissions using RWD and/or external control arms [11, 17, 18]. Like Jahanshahi et al., we found that single-arm trials met greater regulatory acceptance in the context of rare diseases. Similarly, Jaksa et al.'s examination of the influence of external control arms (ECAs) on regulatory decisions and the importance of data quality corresponds closely with our identification of criticisms related to imbalances between ECAs and trial arms, outcome ascertainment bias, and data quality issues. Izem et al.'s focus on the contextualization of single-arm trials using real-world data (RWD) aligns with our study's emphasis on the rare disease context and the utilization of natural history studies as common external controls.

Our results are also consistent with studies noting a general increase and upward trend in the use of RWE in regulatory submissions in both the United States and European Union. In a review of NDA submissions to the FDA from 2019 to 2022, Purpura et al. found a substantial increase in single-arm trials, reported that regulators often flagged issues with endpoint objectivity, and emphasized need for increased guidance for assessing single-arm trials as fit for regulatory submission and approval [14]. In our study, we found that the majority of products approved with pivotal SATs had objective and/or large expected endpoint sizes. This is in alignment with Vaghela et al.’s recent systematic review of FDA-approved non-oncology orphan drug therapies that used RWD, which found increased regulatory acceptance of RWD studies demonstrating a large effect size [67]. Our finding that natural history studies constituted all RWD-based external control arms appears aligns with an earlier study of EMA authorizations and FDA approvals; Flynn et al. found that registries were the most commonly used data source in 2018 and 2019. [15]

Our findings align with the recent FDA guidance, which supports the use of externally controlled trials in rare diseases with well-defined natural histories and limited treatment options. Both our findings and the FDA guidance highlight the importance of high quality patient-level data. Similar to the concerns raised in the guidance, our study noted significant critiques regarding the comparability of ECAs. The high proportion of FDA approvals mentioning SAT data in product labeling mirrors the agency’s emphasis on transparency in presenting efficacy evidence​. While the EMA does not currently have dedicated guidance on externally controlled trials, the ICH E10 guideline on control groups discusses external controls, emphasizing the necessity for appropriate methodological approaches to ensure the validity and reliability of the efficacy data [68, 69]. The 2001 EMA guideline takes a more cautious stance than the FDA despite similar numbers of approvals between agencies in this review. This may suggest a need for updated guidance on externally controlled trials that reflects current European regulatory perspectives. In the absence of updated EMA guidance and relative recency of FDA guidance on SATs and ECAs, our framework provides a useful and succinct summary of key considerations that is consistent with the present regulatory landscape.

Despite the many strengths of this review, there were some limitations. First, while the development of our framework was a phased process conducted with expert input and focus groups, we did not conduct systematic reviews or structured interviews to guide its creation. Instead, we chose to test the framework with a systematic approach for novel approvals in non-oncology as they pose potentially the greatest regulatory challenges to SAT submissions. Further testing of other aspects of this framework to better understand regulatory considerations in other types of submissions.

We were unable to compare results from approvals to applications that were ultimately rejected by the FDA, because these are not publicly available. While the EMA does publish reports on authorization applications that were refused or withdrawn, this was not in scope for this study. Within our study scope, we are unable to pinpoint why certain applications using SATs as pivotal evidence were approved, while others may not have been. In addition, we based our review on unstructured medical and statistical reviewer comments, and some factors may not have been mentioned despite being relevant. Additionally, we did not collect data on the history of communications between the applicant and agency. Future studies would benefit from more detailed monitoring of communications between parties to determine whether aspects in the communication between agency and applicant influence the likelihood of a new drug or biologic application being approved.

We were also limited by differences between how the EMA and FDA review drug applications. The EMA appeared to publish approvals more consistently than the FDA, and EMA approval documents maintained a uniform format, making the analysis of European approvals more systematic. In some instances, the agencies also classified evidence differently. For example, the FDA language described single-arm pediatric extrapolation studies at the time of submission for the first indication, as pivotal evidence while the EMA considered these studies supportive. Due to these differences, we elected to remove studies from our analysis that used single-arm trials exclusively to extrapolate to pediatric patients. Discrepancies both within and across agencies in how trials were presented in review documentation may also have led to bias in the identification of pivotal vs. supportive trials.

Lastly, our analysis had somewhat limited scope. Our exclusion of oncologic indications may limit the generalizability of these results. Most applications covered conditions that were rare (including orphan drugs), had significant unmet medical need, and lacked effective SoC options. Thus, it is difficult to assess if and how single-arm trials and ECAs could be employed for conditions that are more common and have acceptable, if not ideal, SoC therapies. While traditional RCTs could be used to study new therapies for common conditions, it can be difficult to recruit for RCTs if the control arm is not as effective as treatments that are already available. A smaller control arm in the RCT along with a well-constructed ECA could be beneficial and improve efficiency and duration of trials to provide patients faster access to effective medicines. We restricted our review to initial indications, excluding supplemental applications and label expansions. Future studies should consider including approvals outside this time window, therapeutic areas, and submission types to determine if the findings are consistent.

Despite the limitations, this review is the first to directly assess regulatory responses to specific features of single-arm trials submitted as pivotal evidence for product approvals and authorizations. Our study offers the first comprehensive examination of how regulators respond to submissions employing these designs beyond the realm of oncology. This departure from the oncology-focused analyses is particularly robust for two reasons: a) single-arm trials tend to encounter greater regulatory acceptance in oncology submissions, necessitating a distinct evaluation for other therapeutic areas, and b) the landscape of available data for comparison and context differs significantly outside of oncology. Our pre-specified systematic methodology involved scrutinizing all approvals within a specified timeframe, to identify single-arm trials submitted to support approval in filings without RCTs. This methodological approach allowed us to meticulously sift through an extensive volume of regulatory data, to provide a comprehensive understanding of the regulatory landscape surrounding single-arm trials across various therapeutic domains.

Our results are consistent with the medical, regulatory, methodological, and data quality factors identified to affect regulatory acceptance of SATs in our framework. In a fast evolving regulatory landscape in the United States and Europe, our framework provides a summary that is useful early in drug development stages, allowing stakeholders to understand potential regulatory critiques that they may face in using a single arm study for pivotal evidence in non-oncology approvals.

## Conclusion

Based on recent FDA and EMA approvals, the likelihood of regulatory success for SATs with ECAs appears to depend on many design, analytic, and data quality considerations. Our framework is useful in early drug development to guide discussion when considering single-arm trial strategies for evidence generation.

## Supplementary Information

Below is the link to the electronic supplementary material.

Supplementary file1 (DOCX 27 kb)

## Data Availability

No datasets were generated or analysed during the current study.
