# Supplementary material for: A Framework for the Use and Likelihood of Regulatory Acceptance of Single-Arm Trials
Source: Ther Innov Regul Sci. 2024 Sep 16;58(6):1214–32. doi: 10.1007/s43441-024-00693-8 (PMC11530569; doi:10.1007/s43441-024-00693-8)
Supplement: Supplementary file 1 — Supplementary file1 (DOCX 27 kb) [file 43441_2024_693_MOESM1_ESM.docx]

**Appendix Table A1. Selection Criteria for Submissions.**

| **PICOS criteria** | **Inclusion** | **Exclusion** |
| --- | --- | --- |
| **P**opulation | Indicated for a non-oncology population | Oncology, radiologic, or imaging indications, vaccines, and non therapeutic blood products (e.g. blood typing agents) |
| **I**nterventions/Exposures | First approval for a new indication Drugs and Biologics (NDAs and BLAs)  Any approval for which at least one single-arm Phase II or III study was submitted as pivotal evidence | Approvals of devices, generics, biosimilars, or supplements for additional indications  Approvals which did not include any Phase II or III single-arm study as pivotal evidence |
| **C**omparisons | None | None |
| **O**utcome(s) | Concerns or strengths discussed in regulatory review about single-arm design and/or external control arm | Any regulatory review of aspects of supporting study unrelated to single-arm design |
| **S**ource type | FDA and EMA review documentation | Other regulatory bodies |
| **Ti**ming | All approvals between January 1, 2019 and December 31, 2022 | Any approvals outside of this timeframe |

**Appendix Table A2. Full list of fields abstracted for each product from FDA and EMA Review Documentation.**

|  | **Field** | **Data Type** | **Notes on data definition and abstraction process (where applicable)** |
| --- | --- | --- | --- |
| **General submission and approval information** | **Approval ID (unique to this review)** | Numeric |  |
|  | **Product Name (Brand)** | Free Text |  |
|  | **Molecule or Proper Name** | Free Text |  |
|  | **Applicant** | Free Text |  |
|  | **Agency** | Binary: FDA or EMA |  |
|  | **If FDA, CDER or CBER?** | Binary: CDER or CBER |  |
|  | **Drug or Biologic** | Binary: Drug or Biologic |  |
|  | **Application type (EPAR, NDA, BLA)** | Options: EPAR, NDA, BLA |  |
|  | **Application Number** | Numeric |  |
|  | **Date of Approval or Marketing Authorisation** | MMDDYY |  |
|  | **Year of Approval** | YYYY |  |
|  | **Indication** | Free Text | Directly from review documentation |
|  | **Approval includes Pediatric Population?** | Binary |  |
|  | **Priority Review?** | Binary | Directly from review documentation |
|  | **Orphan Designation?** | Binary | Directly from review documentation |
| **Totality of pivotal evidence submitted** | **Single-arm study produced pivotal efficacy evidence** | Binary | Some review documents included tables that specific “pivotal” or “primary” evidence over supportive evidence. For these products, any study that was not listed as supportive were considered pivotal evidence. Where this distinction was not made, we examined review strategy text to determine which trials were not purely supportive evidence In situations where there was only one Phase 2/3 or 3 trial listed at all for efficacy, that was considered pivotal. If the SAT was an extension study or long term safety study, it was considered non-oivotal, unless it was the only Phase 2 or 3 study included in the submission. Any SATs ongoing at the time of submission were considered non-pivotal unless the review strategy text explicitly stated otherwise |
|  | **Single-arm study was submitted in conjunction with traditional well-controlled study/studies as pivotal evidence** | Binary | If any RCT was submitted alongside the pivotal SAT(s), this value was 1. |
|  | **Only pivotal efficacy evidence came from single-arm study or studies** | Binary | If any RCT was submitted alongside the pivotal SAT(s), this value was 0. |
|  | **Single-arm study was used for pivotal evidence in pediatric population only** | Binary |  |
|  | **Single-arm* study had a non-randomized control group, including benchmarks, natural history, past trials, etc (not including baseline controls / pre-/post- comparisions)** | Binary | If any external control arm was used (i.e. not a within study comparison to patient's own baseline), this value was 1 |
|  | **Pivotal single-arm study Phase** | Options: 2 or 3 |  |
|  | **Notes** | Free Text |  |
| **General information on overall reviewer response** | **Review mentions any concern about single arm study design or comparator (e.g. balance on baseline characteristics)** | Binary |  |
|  | **Review mentions any concern about single arm study design or comparator - text** | Free Text |  |
|  | **Review mentions any positive aspect of single-arm study design (e.g. unmet need, large effect size)** | Binary |  |
|  | **Review mentions any positive aspect of single-arm study design - text** | Free Text |  |
|  | **Study includes some external control arm (ECA)** | Binary |  |
|  | **Review mentions any concern about comparator/ECA** | Binary (0 if no ECA) |  |
|  | **Review mentions any concern about comparator/ECA - text** | Free Text |  |
|  | **Review mentions any positive aspect of comparator/ECA** | Binary (0 if no ECA) |  |
|  | **Review mentions any concern about positive aspect of comparator/ECA - text** | Free Text |  |
|  | **Statistical Reviewer recommends approval based on single-arm study (partially or wholly)** | Binary |  |
|  | **Notes** | Free Text |  |
| **Labelling Information** | **Single-arm study included/mentioned in product labeling** | Binary | If the label mentioned a single-arm study by name or references clinical evidence as "uncontrolled", this value was 1 |
|  | **Comparator/ECA included/mentioned in product labeling** | Binary (0 if no ECA or baseline-control) | If language indicates that the single-arm study was compared to anything external (e.g. literature, natural history, past trials), this value was 1. |
|  | **Notes** | Free Text |  |
| **Agency reviewer responses to submission of pivotal SAT(s)** | **Rare disease or gene therapy with inability to recruit placebo** | Binary | Authors' assessment |
|  | **Reviewer notes rare disease or gene therapy with inability to recruit placebo** | Binary | Review documentation must explicitly reference context as rare disease |
|  | **Not rare but inability to recruit placebo** | Binary | Authors' assessment |
|  | **Reviewer notes not rare but unable to recruit placebo** | Binary | Review documentation must explicitly state that recruiting controls or randomizing would be infeasible or unethical |
|  | **Objective primary endpoint** | Binary | Authors' assessment |
|  | **Reviewer notes objective primary endpoint** | Binary | Review documentation must either explicitly state that primary endpoint is objective (1) or explicitly critique a lack of objectivity in the endpoint (0) |
|  | **Discrepancy in assessment of objective endpoint?** | Binary | Indicates whether the paper authors' noted an objective endpoint, but the reviewers did not explicitly state this. |
|  | **Determination of endpoint objectivity** | Binary | In cases where there was a discrepancy, senior reviewer made a final determination. Endpoint was only ruled objective if senior reviewer agreed it was implicitly objective |
|  | **Reason for determination of endpoint objectvitity** | Free text | Documentation of reason for senior reviewer decision |
|  | **Primary Endpoint** | Free text | Abstracted directly from review documentation |
|  | **Effect Size of Primary Endpoint** | Free text | Abstracted directly from review documentation |
|  | **Large effect size in primary endpoint** | Binary | Effect size was considered large if (1) reviewer explicitly stated that effect was large (2) if effect size was large corresponding to Temple 2012 (RR>1.5) |
|  | **Notes on effect size** | Free Text |  |
|  | **Different procedures required for intervention & control** | Binary |  |
|  | **Reviewer notes that different procedures would be required for intervention & control** | Binary |  |
|  | **Established natural history without spontaneous improvement** | Binary |  |
|  | **Reviewer notes established natural history without spontaneous improvement** | Binary |  |
|  | **Severe condition with no effective therapies for control / limited SoC options** | Binary |  |
|  | **Reviewer notes severe condition with no effective therapies for control / limited SoC options** | Binary |  |
|  | **SoC is well established or effective** | Binary |  |
|  | **Reviewer notes that SoC is well established or effective** | Binary |  |
|  | **Unblinded randomization or some other parallel study deisgn is possible** | Binary |  |
|  | **Reviewer notes that unblinded randomization or some other parallel study deisgn is possible** | Binary |  |
|  | **Requires complex safety assessment or active comparator for safety contextualization** | Binary |  |
|  | **Reviewer notes that submission requires complex safety assessment or active comparator for safety contextualization** | Binary |  |
|  | **Reviewer has perception of ability or need to enroll placebo** | Binary |  |
|  | **Language used in review to describe need for single-arm study** | Free Text |  |
|  | **Notes** | Free Text |  |
| **ECA information** | **ECA is person-level** | Binary (0 if no ECA) | This differentiates between aggregate benchmarks (value of 0) and person-level ECAs |
|  | **ECA is from past trial data** | Binary (0 if no ECA) | Corresponds to any data collected in a past clinical trial setting |
|  | **Comparator is patient's own baseline** | Free Text | Corresponds to any endpoints where there is a comparison to a baseline value rather than an external value |
|  | **ECA is from RWD** | Binary (0 if no ECA) | RWD is defined as any data initially collected not for the purpose of the SAT; includes Electronic Health Records, Administrative Claims Data, and Disease Registries/Natural History Studies |
|  | **ECA RWD source 1** | Options: EHR, Claims, Registry or Natural History Cohort |  |
|  | **ECA RWD source 2** | Options: EHR, Claims, Registry or Natural History Cohort |  |
|  | **ECA RWD source 3** | Options: EHR, Claims, Registry or Natural History Cohort |  |
|  | **ECA uses multiple types of RWD** | Binary (0 if no ECA) |  |
|  | **ECA uses RWD linkages** | Binary (0 if no ECA) | Refers to any data where multiple RWD sources are linked at the patient level |
|  | **Notes** | Free Text |  |
| **Agency reviewer responses (critiques and positive assessments) to submission of external control arm,** | **Reviewer notes that comparator is a benchmark / not patient-level** | Binary (Leave blank if no ECA) |  |
|  | **Reviewer notes that patient level data is not accessible by regulator or 3rd party data, ownership limits regulators QA or inspections** | Binary (Leave blank if no ECA) |  |
|  | **Reviewer notes that endpoint measured in ECA is subjective or otherwise unreliable** | Binary (Leave blank if no ECA) |  |
|  | **Reviewer notes that ECA is not contemporaneous to trial** | Binary (Leave blank if no ECA) |  |
|  | **Reviewer notes that ECA data is not generated among geographically representative populations and/ or similar practice setting as single arm trial** | Binary (Leave blank if no ECA) |  |
|  | **Reviewer notes that ECA and trial arms are not balanced on baseline covariates or that comparable inclusion exclusion criteria cannot be applied to both arms** | Binary (Leave blank if no ECA) |  |
|  | **Reviewer notes that study cannot provide a clinical alternative at a similar point in the disease progression as the single arm trial** | Binary (Leave blank if no ECA) |  |
|  | **Reviewer notes that there is risk of outcome ascertainment bias in the comparator arm** | Binary (Leave blank if no ECA) |  |
|  | **Reviewer notes data quality issues** | Binary (Leave blank if no ECA) | Data Quality Issues were identified only if reviewer explicitly flagged this |
|  | **Language used in review to describe ECA** | Free Text |  |
|  | **Notes** | Free Text |  |
